# M^6^A RNA epitranscriptome dynamics linked to major depressive disorder and suicide risk

**DOI:** 10.1038/s41386-025-02165-5

**Published:** 2025-07-10

**Authors:** Bhaskar Roy, Yogesh Dwivedi

**Affiliations:** Department of Psychiatry and Behavioral Neurobiology, Heersink School of Medicine, University of Alabama at Birmingham, Birmingham, AL USA

**Keywords:** Depression, Epigenetics and behaviour

## Abstract

Major depressive disorder (MDD) is the most prevalent psychiatric disorder. MDD patients are at substantially increased risk of dying by suicide. The molecular mechanisms associated with MDD and associated suicide are not clearly understood, which impedes the development of novel therapeutics. N6-methyladenosine (m6A) is the most prevalent epitranscriptomic mark on mRNA and plays significant roles in various physiological processes. This study investigated m6A RNA methylation and its potential contributions to MDD pathogenesis and associated suicide risk. High-throughput microarray analysis in the dorsolateral prefrontal cortex (dlPFC) of MDD subjects (*n* = 49) and non-psychiatric controls (*n* = 49) identified 1290 significantly hypermethylated and 6842 hypomethylated transcripts, with most m6A sites enriched in coding sequences. Chromosome-wide analysis showed hypermethylation hotspots on chromosomes 1 and 19. In-silico analysis identified enriched AAGA and ACCCA m6A motifs in the MDD group. Expression analysis revealed reduced FTO demethylase and increased METTL3 methyltransferase levels. A majority of M6A hypermethylated genes showed inverse correlation with their expression levels. Functional enrichment of hypermethylated genes highlighted disruptions in molecular pathways relevant to MDD. Comparison of MDD-non-suicide (*n* = 32) and MDD-suicide (*n* = 17) identified 6750 transcripts with significant hypermethylation, whereas 6159 transcripts had significant hypomethylation in the MDD-suicide group; of them, 196 hypermethylated genes were explicitly associated with suicide in the MDD group, whereas 38 hypermethylated genes appeared to elevate suicide risk in MDD patients. Also, the MDD-suicide group had distinct neuromolecular pathways associated with these risk genes. Collectively, the findings suggest a critical role for m6A methylation in modulating the molecular networks underlying MDD and suicide susceptibility.

## Introduction

Major depressive disorder (MDD) is the leading cause of disability worldwide and is characterized by persistent sadness, lack of interest, and cognitive impairment. MDD is ranked third in terms of disease burden, and it is predicted to rank first by 2030 [1]. Despite significant research efforts, the underlying neurobiology of MDD remains poorly understood. The currently widely accepted theories of MDD pathogenesis include the neurotransmitter and receptor hypothesis, the hypothalamic-pituitary-adrenal (HPA) axis hypothesis, and the neuroimmune hypothesis. However, these hypotheses cannot fully explain the pathological mechanisms of MDD. Since a significant proportion of MDD patients do not respond to currently available medications, and recent studies have shown that the severity of illness is a major predictor of nonadherence [2] especially in patients with mood disorders—alternative theories are being proposed. Among these are gene-environment interactions and epigenetic modifications of genes that are critical to the brain’s adaptability to adverse events in both early and late life [3, 4]. These vulnerabilities are further enhanced with increased psychosocial-related issues during vulnerable periods [5].

Recently, a paradigm-shifting phenomenon has been introduced with the unique concept of RNA regulation through an “epitranscriptomic” mechanism [6, 7]. Part of this comes from methylation changes of RNA transcripts at the sixth position of adenosine (m6A) residues [8]. M6A modification of RNA occurs primarily on the conserved DRACH sequence motif (D = A/G/U, R = A/G, H = U/A/C) and is the most prevalent endogenous mRNA modification in mammals [8]. It occurs at tens of thousands of sites throughout the transcriptome, with a frequency of 0.15–0.6% of all adenosines [9]. The m6A methylation on mammalian coding transcripts are well-orchestrated and is subjected to precise regulation. This occurs through “writers” (METTL3, METTL14, Wilm’s Tumor-1-associated Protein [WTAP]), which methylate the adenosine at the N6 position; “erasers” (Fat mass and obesity associated protein [FTO], alkB homolog 5 [ALKBH5]), which catalyze the reversible oxidative demethylation of the methyl group from m^6^A; and “readers” (YTH domain family of proteins, YTHDF1 and YTHDF2) that are a group of effectors that determine the fate of modified mRNAs by binding to specific RR(m6A)CH sequence in m^6^A sites [8]. M6A methylation can alter RNA metabolism by changing RNA structure, splicing RNAs, or regulating mRNA maturation, therefore promoting either translation or accelerating mRNA decay [8]. Since M6A methylation is a highly dynamic and reversible epitranscriptomic marker, examining its changes in the MDD brain might uncover how transient shifts in gene expression may contribute to depression [10, 11]. This may also offer new insights into MDD as a disorder of molecular plasticity [12].

Because m6A is reversible, dynamic, and the most prevalent type of mRNA modification in the brain, it has the potential to regulate gene expression changes influenced by environmental stimuli [13]. It has been reported that altered m6A methylation in neurons is associated with increased fear memory and a changed transcriptome response to fear and synaptic plasticity [14, 15]. In the context of MDD, abnormal m6A methylation patterns have been implicated in the dysregulation of genes critical for neuroplasticity, synaptic functions, and the stress response [16, 17]. We and other researchers have shown altered m6A methylation in brain-derived neurotrophic factor (BDNF) transcripts, which play a crucial role in synaptic plasticity and have been linked to MDD [18, 19]. Similarly, changes in m6A patterns affecting the serotonergic system, including serotonin transporter and serotonin receptor isoforms, suggest that m6A may influence neurotransmitter balance and mood regulation [16]. Recent studies have linked genetic polymorphism of *FTO* and *ALKBH5* genes to MDD development [20]. Other findings also indicate that m6A methylation-associated changes in gene functions control the morphological attributes of neurons, such as axonal and dendritic growth [21].

Whereas the above studies have provided insights into understanding their role in MDD, there remains a critical gap in understanding how m6A methylation changes in the brain could potentially contribute to MDD pathogenesis. Given the critical role of m6A RNA methylation in regulating gene expression, we hypothesize that alterations in m6A modification and the activity of m6A-modifying enzymes could contribute to the pathogenesis of MDD by influencing genes involved in mood regulation, stress response, and neural plasticity. This study aimed to examine the role of m6A RNA methylation in MDD by employing a high-throughput m6A-specific expression microarray to profile the m6A methylation landscape across 56,618 RNA transcripts and associated functions in a large cohort of MDD and non-psychiatric control subjects in the dorsolateral prefrontal cortex (dlPFC), a brain area highly relevant to emotion, cognition, and mood regulation. In a secondary analysis, we also distinguished molecular signatures associated with suicide risk among MDD individuals. Our findings not only revealed large-scale changes in m6A methylation but also m6A-methylation-mediated disruptions in synaptic signaling, neurotransmitter transport, and neuroimmune regulation. Additionally, the MDD-suicide group exhibited distinct epitranscriptomic changes and associated neuromolecular pathways. Altogether, our study adds a critical dimension to the present understanding of the gene regulatory mechanisms and how this could potentially play a role in MDD and suicide pathogenesis.

## Materials & methods

The detailed methods used in this study are provided in the Supplementary section.

### Subjects

The study was performed in the dorsolateral prefrontal cortex (dlPFC) from 49 nonpsychiatric controls (referred to as controls) and 49 MDD subjects obtained from the Maryland Psychiatric Brain Collection. Within the MDD group, there were 17 MDD-suicide (MDD-S) subjects and 32 MDD-non-suicide (MDD-NS) subjects. Demographic and clinical data are shown in Supplementary Table S1. The study was approved by the IRB of the University of Alabama at Birmingham. Detailed psychological autopsy procedures and brain dissection are provided in the Supplementary section.

### M^6^A methylation enrichment analysis following m^6^A mRNA immunoprecipitation (MeRIP)

Total RNA was extracted from dlPFC, purified, and fragmented before being used for m6A methylation profiling. RNA integrity and absence of DNA were confirmed by Bioanalyzer RNA Nano chips (Agilent Technologies, USA). Samples with RIN > 7 were used for further analysis. M6A-modified RNA was enriched using anti-m6A antibody-based immunoprecipitation (MeRIP), followed by elution and purification for downstream analysis.

### Microarray hybridization and analysis

A microarray expression profiling was performed to capture m6A-based RNA modifications by hybridizing Cy5-labeled (IP) and Cy3-labeled (Sup) cRNAs onto a human epitranscriptomic microarray. The acquired array images were analyzed using the Agilent Feature Extraction software (version 11.0.1.1). Raw intensities for the “IP” (Cy5-labeled) and “Sup” (Cy3-labeled) samples were normalized with the average log2-scaled intensities of the spike-in RNA controls. The m6A methylation level was calculated based on the normalized Cy5-labeled “IP” intensities. The raw intensities of Cy5-labeled and Cy3-labeled RNAs were normalized with the average log2-scaled spike-in RNA control intensities. The m6A methylation percentage was calculated based on the normalized intensities of the Cy5- and Cy3-labeled RNAs. Fold change and *p*-values were determined for each transcript between the comparison groups. Differentially m6A-methylated RNAs were identified by applying a fold change (FC) cutoff of ≥1.5 or ≤0.7 and a *p*-value threshold of <0.05. The differential changes were then adjusted for multiple testing using the Benjamini–Hochberg false discovery rate (FDR) correction method [22]. Lastly, hierarchical clustering was performed to visualize the differential m6A-methylation patterns among the samples.

### RNA sequencing-based transcriptome profiling

RNA sequencing-based expression profile was performed to analyze transcriptome-wide changes in the dlPFC using poly(A)-selected or rRNA-depleted RNA. Libraries were sequenced on an Illumina HiSeq4000, and differential gene expression was assessed through quality-controlled alignment, FPKM quantification, and statistical analysis with ballgown and iDEP tools. Briefly, raw data files in FASTQ format were generated from the Illumina sequencer. To examine the sequencing quality, the quality score plot of each sample was plotted and examined using the FastQC software. After quality control, the fragments were 5’, 3’-adaptor trimmed and filtered ≤20 bp reads with cutadapt software. The trimmed reads were aligned to reference genome with Hisat 2 software. Based on alignment statistical analysis (mapping ratio, rRNA/mtRNA content, fragment sequence bias), results were used for subsequent data analysis. The expression levels (FPKM value) of known genes and transcripts were calculated using ballgown through the transcript abundances estimated with StringTie. The number of identified genes/groups was calculated based on the mean FPKM (*P* ≥ 0.5). Heatmap and k-means clustering were visualized for the expressed genes using the iDEP tool suit 25. Differentially expressed gene analysis was performed with the R package ballgown. Expressed genes were used to create Volcano plots using R (v.3.6.3) library. The threshold for the *p*-value cutoff of the expressed gene was assigned ≤0.05. Up- and down-regulated genes are depicted by red and green color dots, respectively. The remaining insignificant genes are depicted as dark gray dots. The x- and y-axes correspond to the log2 fold change value and the mean expression value of log 10 (*p*-value), respectively.

### qPCR expression of M6A modifying enzymes

To assess the expression of m6A-modifying enzymes, cDNA was synthesized from 500 ng RNA using M-MLV reverse transcriptase and oligo(dT) primers, followed by qPCR with gene-specific primers (Supplementary Table S2). Relative expression levels were quantified using EvaGreen dye and the ΔΔCt method, with GAPDH as the internal control.

### Gene ontology (GO) and functional clustering of M6A-enriched coding transcripts

GO enrichment of m6A-enriched transcripts was performed using DAVID and visualized via ClueGO in Cytoscape, with GO Term Fusion applied to refine redundancy. Statistically significant GO terms (*P* < 0.05) were plotted using ggplot2 and SynGO for synapse-specific enrichment, based on a custom background of expressed genes.

### RNA expression vs. M6A methylation correlation analysis

Pearson correlation analysis was conducted to evaluate the relationship between m6A methylation enrichment and mRNA expression levels. Correlation coefficients (R) and *p*-values were calculated to determine the strength and significance of the association.

### Statistical analysis

Statistical analyses were conducted using SPSS (v.29, IBM, USA). The Shapiro–Wilk test was used to assess the normality of the data. The average difference of age, PMI, and brain PH was assessed by the Student’s *t* test. Differences in gender, drug abuse, alcohol abuse, and antidepressant toxicology were analyzed by Fisher’s exact test. The average difference in gene expression was compared by the Student’s *t* test. The correlation between the fold change of RNA-seq and qPCR was calculated with the Pearson correlation coefficient. Correlations of the gene expressions with covariates were also conducted with the Pearson correlation coefficient. Statistical significance was set at the 95% level (*p* ≤ 0.05).

## Results

### Transcriptome-wide M6A RNA methylation profile and feature extraction of M6A methylated transcripts in the dlPFC of MDD and control subjects

**Fig. 1:**
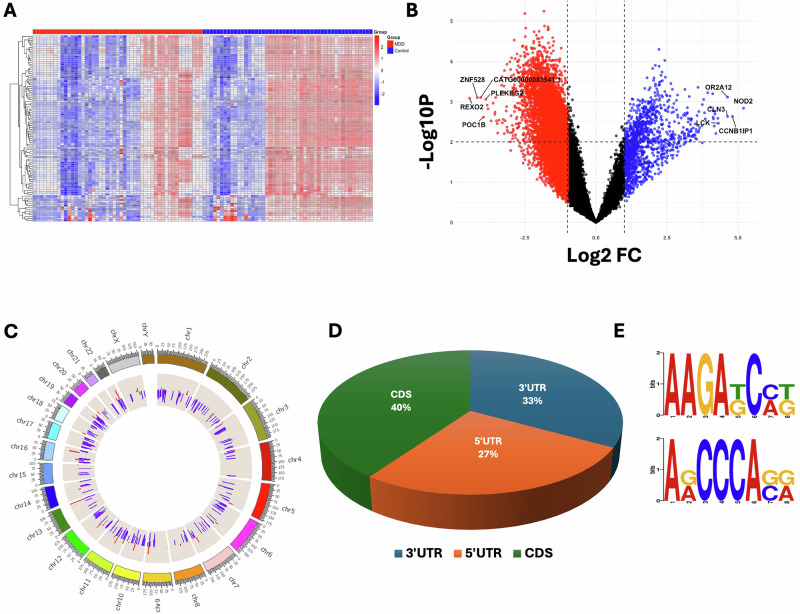
MDD-associated m6A RNA methylation profiling and annotation in dorsolateral prefrontal cortex following microarray. **A** An expression heatmap showing normalized methylation enrichment values of RNA transcripts was determined across samples based on a group-wise comparison between control subjects (*n* = 49) and MDD subjects (*n* = 49). A cluster dendrogram has been added by clustering the 100 differentially methylated transcripts across the samples based on their hierarchical clustering following similarities in their expression pattern. **B** Volcano plot from microarray m6A methylation data analysis. Red and blue dots indicate hypomethylated and hypermethylated RNA transcripts from microarray methylation data analysis results. **C** Circular plot showing chromosome-wise annotation of differentially methylated transcripts and their expression profile in MDD (data presented as log2FC). In the plot, individual tracks or rings represent different annotations. The outermost track displays the cytoband for human reference genome version hg38. The following two tracks display hyper (red) and hypo (blue) methylated transcripts. The outermost periphery of the circos plot represents chromosome number. **D** MDD-specific percent-wise distribution of m6A sites on coding transcripts is shown in the pie chart. As mapped in the chart, primarily the m6A sites were associated with the coding region (CDS) of mRNA transcript, flanked by 5’ and 3’ untranslated regions (UTRs). **E** Consensus motifs associated with m6A site enrichment on RNA transcripts. Two motifs were mapped with slight difference at the 3^rd^, 4^th^, and 8^th^ nucleotide positions.

We determined the differential m6A methylation changes between MDD and control subjects and filtered the results based on statistical significance (*p* ≤ 0.05). An overall m6A methylation profiling in control and MDD subjects is provided in Supplementary Table S3. In the MDD group, 4869 transcripts were hypermethylated and 15,741 hypomethylated compared to the control group, irrespective of significance. However, when considering *p* ≤ 0.05, 1290 transcripts exhibited significant hypermethylation, and 6842 showed significant hypomethylation. Significantly hyper- and hypomethylated transcripts (gene symbols), along with their fold changes and significance levels, are listed in Supplementary Tables S4 and S5. Methylation changes are visualized in a heatmap of the top 100 differentially regulated m6A methylation changes (Fig. 1A), and the differential methylation profile between the control and MDD groups is shown in a volcano plot (Fig. 1B). In the volcano plot, regardless of statistical significance, the distribution shows a significantly higher number of hypomethylated transcripts (15,741, red) compared to hypermethylated ones (4869, blue) in the MDD group.

To better understand the distribution of m6A methylation sites across the genome, significant hypermethylated (FC ≥ 2) and hypomethylated (FC ≤ 0.5) m6A sites were mapped across all 23 autosomes and the two sex chromosomes (X and Y) in a control vs. MDD comparison. Circular chromosomal plots (Fig. 1C) revealed hypermethylation across all chromosomes, especially on chromosomes 9, 13, 16, and 22. Notably, chromosomes 1 and 19 had the highest density of hypermethylated gene transcripts (22 genes on chr 1, 19 genes on chr 19), while chromosomes 13, 18, and 21 had fewer, with chr 21 having only 2. Additionally, 40% of methylation sites were linked to the coding DNA sequence (CDS) of mRNA transcripts, compared to 27% in 5’ untranslated region (UTR) and 33% in 3’ UTR (Fig. 1D). In-silico motif analysis of all methylated transcripts in the MDD group further revealed the enrichment of the m6A consensus motif (RRACH) in all samples. Two high-confidence consensus motifs, AAGA and ACCCA, were identified based on sequence conservation patterns, both associated with m6A methylation enrichment (Fig. 1E).

### Differential expression of key genes regulating the m6A methylation process in MDD

**Fig. 2:**
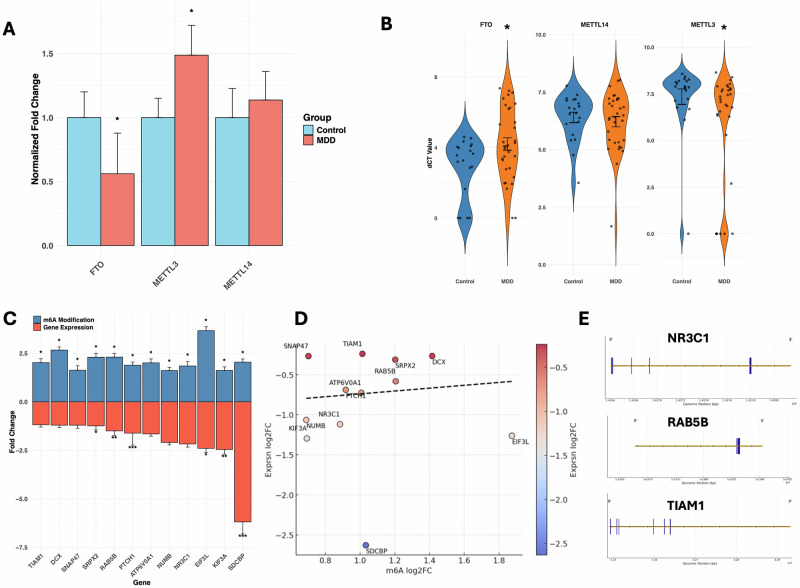
MDD-specific expression profiling of m6A modifying enzymes in dorsolateral prefrontal cortex of MDD and control subjects following qPCR and their relationship with methylation changes. **A** Colored bar plot displaying normalized fold change expression values of key demethylating (FTO) and methylating (METTL3 and METTL14) transcripts in the dlPFC of control and MDD subjects. The expression values of individual genes were normalized with the GAPDH expression values. Data has been presented with standard error of mean (SEM) and significance (*p* ≤ 0.05) between the two groups was determined following Student’s *t* test. **B** Colored violin plot showing expression changes for the same three gene transcripts (FTO, METTL3 and METTL14) but with normalized delta Ct (dCt) calculation. The individual violin plots with dotted scatter diagram showing normalized Ct value distribution across control and MDD samples. The level of significance between the groups has been shown at the top of the respective gene violin plot with the determined *p*-value changes. **C** The inverse correlation between expression level (Fold-Change) of 12 coding gene transcripts and their respective m6A methylation level (Fold-Change) has been presented with an inverted bar plot. The asterisk symbol has been added to the respective gene bar plot to highlight their level of significance for both m6A methylation and gene expression changes (*p < 0.05; **p < 0.005, and ***<0.0005). **D** The Pearson correlation analysis of m6A methylation vs gene expression of the 12 impacted gene transcripts has been presented with a scatter plot. The dashed regression line showing the inverse correlation between m6A methylation and gene expression changes for the 12 coding transcripts in the MDD group. Both the changes are presented with log2Fold-Change (log2FC) scale to determine the correlation. **E** RNA m6A methylation patterns across the transcript length of select genes (NR3C1, RAB5B, and TIAM1), which are relevant to CNS functions. The enriched methylation sites are depicted as colored bars on a linear transcript diagram, with their corresponding genomic positions shown in base pairs (bp).

To investigate the mechanisms underlying altered m6A methylation in MDD, we analyzed the mRNA expression of key m6A regulatory genes, including FTO, METTL3, and METTL14, in the MDD group. As shown in Fig. 2A, FTO expression was significantly reduced (*p* = 0.03), while METTL3 was upregulated (*p* = 0.04). METTL14 showed no significant difference (*p* = 0.56). Figure 2B displays violin plots with scatter diagrams illustrating expression changes of the same three genes based on normalized delta Ct values.

### Analysis of significantly hypermethylated transcripts and the regulatory influence on their expression status in MDD

We next determined 20 significantly hypermethylated gene transcripts in the MDD group, as these genes have a significant role in pre-, post-, exo-, and extra-synaptic regulation and in modulating the synaptic assembly and anterograde dendritic transport. These included RAB5B, EFNB1, SDCBP, SNAP47, SLC3A2, PLAT, TIAM1, NR3C1, NUMB, LZTS1, CHD4, EIF3L, HNRNPL, ATP6V0A1, PTCH1, SAMD4A, DCX, SRPX2, DMTN, and KIF3A. A list of these 20 genes, along with their corresponding methylation fold-change and significance levels, is provided in Supplementary Table S6. Next, we determined their expression status from RNA-seq data in the same subjects used for m6A methylation. The differential expression analysis identified significant downregulation of 12 out of 20 genes (TIAM1, DCX, SNAP47, SRPX2, RAB5B, PTCH1, ATP6V0A1, NUMB, NR3C1, EIF3L, KIF3A, SDCBP) in the MDD group. Their m6A methylation status and corresponding expression profile are displayed in an inverted bar plot (Fig. 2C). The results showed a strong inverse correlation between the m6A methylation and gene expression profile following the Pearson correlation analysis (Fig. 2D). A detailed m6A methylation and expression fold changes of these 12 genes are presented in Supplementary Table S7. Mapping of the m6A methylation enrichment for some of the genes listed above (NR3C1, RAB5B, TIAM1) showed highly enriched methylation sites (as colored bars) along with their corresponding genomic positions (in base pairs) (Fig. 2E).

### Functional analysis of differentially methylated transcripts in MDD group following methylation correlation, hub identification, gene ontology (GO), pathway, and protein-protein interaction (PPi) analyses

**Fig. 3:**
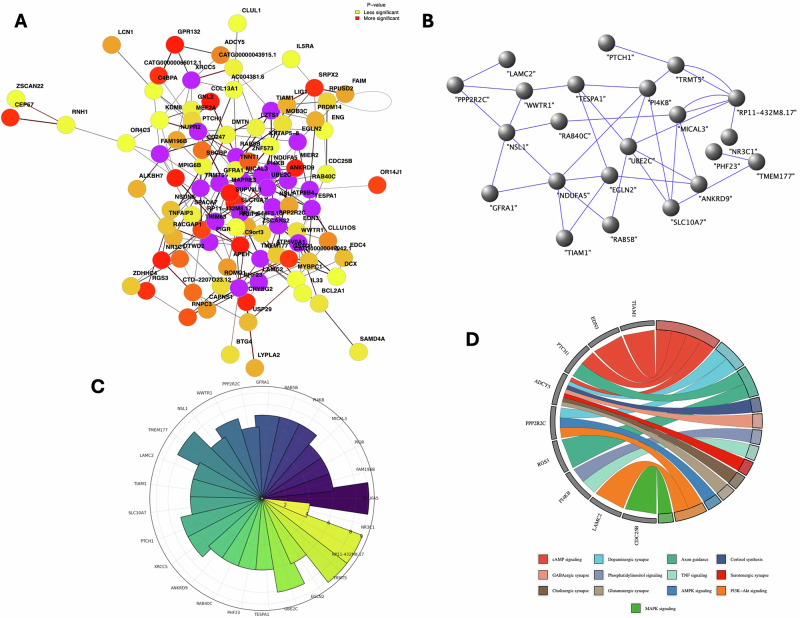
Functional network analysis of hypermethylated gene transcripts and their role in MDD-related pathways. **A** Network connectivity map of the most significantly hypermethylated gene transcripts, with nodes color-coded based on *p*-value (*p* ≤ 0.05) changes and edges representing methylation degrees. The functional relationships between gene transcripts serve as background enrichment, highlighting interactions from highest to lowest statistical significance. **B** A mapped subnetwork is centered around the hub methylated transcripts, which form the core of the functional connectivity network, showing the primary drivers of network functionality based on their interaction patterns. **C** Sunburst plot depicting the hub gene transcripts and their connection scores, providing a visual representation of their centrality and interaction strengths within the network. **D** Colored chord plot showing core genes associated with dysregulated KEGG pathways (such as cAMP signaling, dopaminergic signaling, and others) in the dorsolateral prefrontal cortex of MDD subjects, emphasizing the selective functional enrichment of the top methylated transcripts.

We aimed to investigate whether the most significantly hypermethylated gene transcripts were functionally related and identify the key drivers that maintain higher-order connectivity in the cellular context. To do this, we constructed a network map (Fig. 3A), ranking interactions by statistical significance. In the network, nodes were color-coded based on *p*-value changes (dark red to light yellow), and edges reflected the degree of methylation. Highly significant hypermethylated genes, such as ANKRD9, SLC10A7, SUPV3L1, NR3C1, C4BPA, and APEH, were centrally located, while others like FAM196B, TNFAIP3, EGLN2, LCN1, DCX, SAMD4A, and CLUL1 were arranged based on decreasing significance (light orange to yellow). Hub methylated transcripts (purple circles), including UBE2C and RAB40C, MIKAL3, PI4KB, and NSL1 were primary drivers and more frequently connected with others, forming denser subnetworks (Fig. 3B). Hub gene transcripts and their connection scores are also shown in a sunburst plot (Fig. 3C).

To further explore the selective functional enrichment of the top methylated transcripts used in the network mapping, we queried the KEGG pathway database and filtered for pathways (cAMP signaling, dopaminergic signaling, axon guidance functions, cortisol synthesis, GABAergic signaling, phosphatidylinositol signaling, TNF signaling, serotonergic signaling, cholinergic synapse signaling, glutamatergic synapse, AMPK signaling, PI3K-AKT signaling, and MAPK signaling) known to be dysregulated in MDD. We then presented the core genes associated with these pathways in a colored chord plot in Fig. 3D. The genes included TIAM1, EDN3, PTCH1, ADCY5, PPP2R2C, RGS3, PI4KB, TNFAIP3, LAMC2, and CDC25B. The figure legend provides details on the color codes used to link the genes to their respective pathways.

**Fig. 4:**
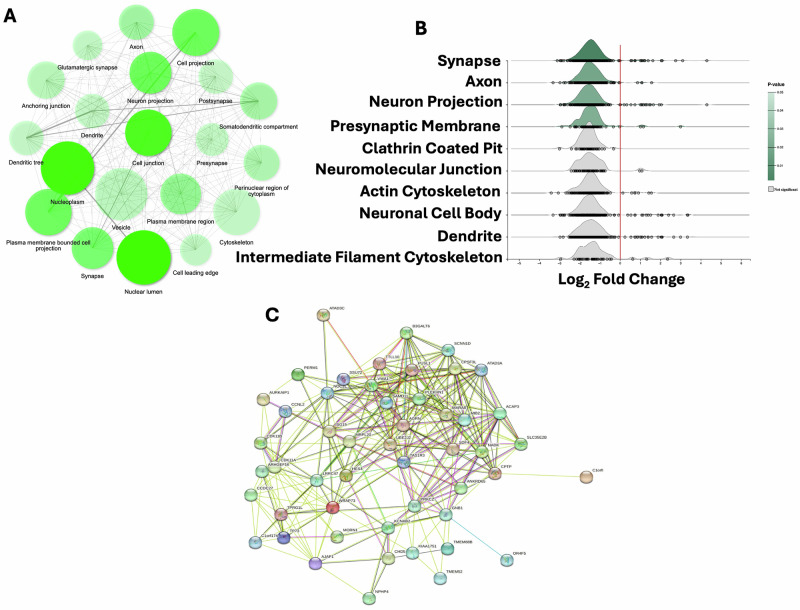
Functional clustering and analysis of significantly hypermethylated transcripts in dorsolateral prefrontal cortex of MDD subjects. **A** Gene ontology (GO) enrichment network plot showing the significantly impacted ontological functions associated with all significantly hypermethylated gene transcripts in MDD brain. The network was based on the betweenness connection to join the most related gene function nodes. The increasing and decreasing strength of connection between the two associated nodes is shown by a thick or thin line of edges. **B** The ridgeline plots display enrichment in various neuronal functions based on their connection to the coding gene with a significantly hypermethylated state in MDD brain. **C** PPi (protein-protein interaction) network based on the direct (physical) and indirect (functional) associations of significantly hypermethylated coding transcripts (in MDD) as obtained from STRING database. The network showing only those methylation-impacted genes that fulfilled the network joining criteria set at the STRING database.

We performed GO enrichment analysis on the most significantly hypermethylated transcripts to understand the functional impact of m6A methylation changes. A comprehensive list of GO terms and associated genes is provided in Supplementary Table S8. The GO enrichment analysis uncovered significant functional disruptions in synaptic signaling, neurotransmitter transport, and the stress response in MDD. GO network analysis (Fig. 4A) identified enriched biological processes related to synaptic activity, vesicle dynamics, presynaptic and postsynaptic organization, and neuroimmune interactions. Key hypermethylated genes involved in these processes included RAB5B (synaptic vesicle trafficking), EFNB1 and SDCBP (presynaptic assembly), and SNAP47 (neurotransmitter release). Ridgeline plots (Fig. 4B) confirmed the localization of these methylation changes, showing enrichment in synaptic vesicles, dendrites, axons, and postsynaptic densities. Further investigation into neuronal structure-related GO terms (Supplementary Table S9) revealed hypermethylation in genes associated with dendritic spine morphogenesis and synaptic plasticity. The ridgeline plots also showed enrichment in axon-related processes and presynaptic membrane architecture, supporting findings of hypermethylation in genes regulating vesicle recycling and neurotransmitter transport. Interestingly, specific methylation changes were localized in genes related to clathrin-coated pits, crucial for synaptic vesicle endocytosis and membrane recycling. This was consistent with the protein-protein interaction (PPI) network analysis (Fig. 4C), which highlighted hypermethylation in synapsin genes (SYN1, SYN2), key regulators of synaptic vesicle mobilization, and neurotransmitter release. Moreover, the ridgeline plots demonstrated that methylation changes were enriched in cytoskeletal components, including genes related to the actin cytoskeleton and intermediate filament cytoskeleton. Hypermethylation was found in genes associated with cytoskeletal stability and remodeling, processes that are essential for neuronal morphogenesis, synaptic architecture, and intracellular transport. These findings align with GO terms associated with neuronal structure, suggesting that altered m6A methylation of certain key genes (as highlighted previously) may impact dendritic spine formation and cytoskeletal dynamics, leading to impaired synaptic plasticity and connectivity. GO terms related to the stress response and neuroinflammatory pathways, including NF-κB signaling, interferon signaling, cytokine stimulus, and neuroinflammatory signaling pathways, were also enriched, with hypermethylation detected in immune-related genes such as IL6, TNF, NF-κB, and NLRP3, as detailed in Supplementary Table S10. The PPI network analysis (Fig. 4C) further highlights central nodes involving proteins critical for synaptic function and neuroimmune regulation, including synapsins (SYN1, SYN2) PSD-95, and inflammatory mediators such as NF-κB and IL-6. Overall, the cohesive distribution of hypermethylated transcripts across synaptic structures, neuronal projections, cytoskeletal components, and stress-related pathways in the ridgeline plots was consistent with the GO network and PPi analyses, supporting the role of m6A RNA methylation in synaptic dysfunction, neuronal connectivity deficits, and neuroimmune alterations in MDD.

### M6A transcriptomic profiling in MDD-suicide

**Fig. 5:**
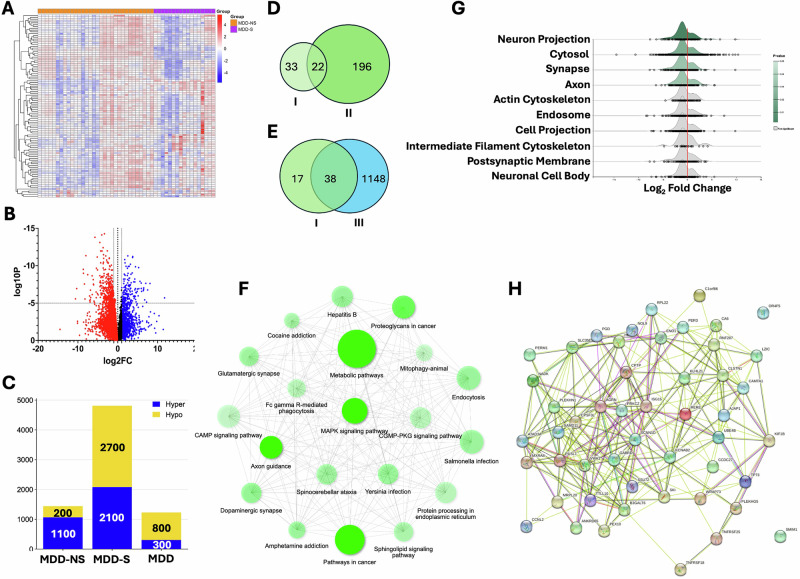
MDD and suicide-associated m6A RNA methylation profiling and functional annotation in the dlPFC following microarray analysis. **A** Expression heatmap displaying normalized methylation enrichment values across samples, comparing MDD non-suicide (MDD-NS; *n* = 32) and MDD suicide (MDD-S; *n* = 17) subjects. A cluster dendrogram was generated using 100 differentially methylated transcripts based on hierarchical clustering. **B** Volcano plot from m6A microarray methylation analysis showing differentially methylated transcripts between MDD-NS and MDD-S groups. Red and blue dots indicate hypo- and hypermethylated transcripts, respectively. **C** Stacked bar diagram showing distribution of hyper- and hypomethylated transcripts across MDD-NS, MDD-S, and combined MDD (MDD-NS + MDD-S) groups. **D** Venn diagram illustrating overlap of significantly hypermethylated transcripts (FC ≥ 2) between Control vs MDD and Control vs MDD-NS comparisons. A total of 33 genes were unique to MDD, 196 exclusive to MDD-NS, and 22 shared. **E** Venn diagram showing transcript overlap between Control vs MDD and Control vs MDD-S groups, with 17 genes unique to MDD, 1,148 unique to MDD-S, and 38 shared. **F** GO enrichment network highlighting significantly affected functional terms based on inter-node betweenness and edge strength. **G** Ridgeline plots displaying enrichment of neuronal functional categories linked to hypermethylated coding genes in MDD-S. **H** PPi network showing direct and indirect associations among hypermethylated transcripts in MDD-S, derived from STRING database criteria.

Since the MDD group included individuals who died by suicide and those who died from other causes, we investigated whether m6A RNA methylation changes could help interpret the heightened risk of suicide within the MDD group. For this, we divided the MDD subjects into MDD-non-suicide (MDD-NS, *n* = 32) and MDD-suicide (MDD-S, *n* = 17) groups. The differential m6A RNA methylation profile between the MDD-S and MDD-NS groups is shown in a volcano plot (Fig. 5A). Overall, 17,608 transcripts were hypermethylated and 14,540 hypomethylated in the MDD-S group compared to MDD-NS, irrespective of significance. However, with *p* ≤ 0.05, 6750 transcripts showed significant hypermethylation and 6159 significant hypomethylation in MDD-S (Supplementary Tables S11 and S12). Methylation changes are also visualized as a heatmap of the top 100 m6A changes (Fig. 5B). The heatmap shows that the MDD-NS group is generally hypermethylated, while the MDD-S group trends toward hypomethylation, highlighting methylation’s role in differentiating the groups.

A ratio of hyper- and hypomethylated sites across the MDD, MDD-S, and MDD-NS groups is shown in a stacked bar plot (Fig. 5C). The MDD-S group exhibited a notable increase in both hypermethylated and hypomethylated sites compared to MDD-NS and MDD groups. To further determine significantly hypermethylated transcripts associated with MDD or suicide, we focused on gene transcripts with a fold change (FC ≥ 2) in three comparisons: Control vs. MDD (I), Control vs. MDD-NS (II), and Control vs. MDD-S (III). Pairwise comparisons of these gene lists revealed unique and shared genes for each group. In the I vs. II comparison, 33 genes were unique to MDD, 196 to MDD-NS, and 22 were shared. In the I vs. III comparison, 17 genes were unique to MDD, 1148 to MDD-S, and 38 were shared. These comparisons, shown in Venn diagrams (Fig. 5D, E), helped identify gene sets specific to MDD, suicide risk, or shared between both. A list of genes from these comparisons is provided in Supplementary Table S13. The analysis revealed that 196 genes uniquely hypermethylated in the MDD-NS group were associated with suicide risk (Supplementary Table S14), while 38 shared genes between MDD and MDD-S were linked to increased suicide risk in MDD patients.

### Functional characterization of M6A methylated transcripts in the MDD suicide group

The GO network analysis comparing the MDD-NS and MDD-S groups revealed distinct functional pathways linked to suicide risk. As shown in Fig. 5F, both groups exhibited enrichment in synaptic function and neuroinflammatory pathways; however, the MDD-S group displayed unique enrichment in pathways related to neuronal development and immune response. Our findings indicate that several key pathways, including axon guidance and MAPK signaling, showed differential m6A methylation changes, affecting genes such as SEMA5A, ROBO2, SHH, PIK3CA, and CAMK2B. These genes are crucial for neuronal connectivity and signal transduction. Additionally, neurotransmitter-associated pathways, particularly those related to glutamatergic and dopaminergic synapses, were significantly enriched with hypermethylated genes. Importantly, the methylation enrichment of genes like GRIK4, GRM1, CACNA1C, and ADCY2 within glutamatergic and dopaminergic pathways may critically impact neuronal excitability and synaptic efficiency, thus altering synaptic transmission in individuals at risk for suicide. Further analysis of cellular components (Fig. 5G) revealed significant m6A enrichment in transcripts associated with the postsynaptic density, essential for synaptic plasticity and signal transduction. Moreover, unique methylation patterns in mitochondrial and endoplasmic reticulum-associated transcripts suggested disturbances in cellular metabolism and stress response.

The PPi analysis in Fig. 5H highlights unique interactions between stress response and neurotrophic proteins, such as CRF and BDNF. Genes involved in GABAergic and inflammatory signaling (GABRB3, RAF1, TNF) showed significant hypermethylation, suggesting altered stress adaptation and neuroinflammation. Methylation changes in calcium signaling genes such as CACNA1A, CAMK2G, and PRKCA indicate impacts on synaptic activity and neuronal plasticity. These findings suggest that m6A methylation changes in the MDD-S group contribute to dysregulated neurotransmission, neuroinflammation, and impaired stress-related circuits, shedding light on suicide risk mechanisms.

### M6A methylation differences between violent and non-violent suicide methods and identifying unique epitranscriptomic signatures associated with violent suicide

The composition of our MDD suicide cohort included a mixed group of violent (*n* = 9) and non-violent (*n* = 8) suicide completers, which prompted us to explore potential differences in m6A RNA methylation between these two groups. Differential expression analysis, followed by a *t*-test, identified a total of 31 significantly hypermethylated gene transcripts in violent suicide completers. Conversely, 131 gene transcripts were found to be hypomethylated in violent suicide completers, meeting statistical significance at *p* < 0.05. All significantly hyper- and hypomethylated gene transcripts, along with their expression fold change and log₂FC values, are listed in Supplementary Table S15. To identify a potential unique epitranscriptomic signature at the level of m6A RNA methylation, differentiating violent suicide from overall suicide risk, we compared the 31 hypermethylated transcripts from the violent group with all significantly hypermethylated genes identified in the total MDD-suicide (MDD-S) cohort. This comparison revealed 20 non-overlapping hypermethylated gene transcripts (Supplementary Table S16), representing a unique m6A epitranscriptomic signature in violent suicide completers.

### Effect of confounding variables on M6A RNA methylation genes

Age, PMI, RIN, and brain pH were not significantly correlated with the top 25 significantly m6A methylated genes in the MDD group (Supplementary Fig. S1). Similarly, race, sex, antidepressant toxicology, and substance and alcohol use had no significant effects on m6A methylated genes except (Supplementary Figs. S2–S6).

## Discussion

Our study revealed significant alterations in m6A methylation in individuals with MDD, which were associated with disruptions in synaptic signaling, neurotransmitter transport, and neuroimmune regulation. Interestingly, the MDD-suicide group exhibited a distinct epitranscriptomic signature, with unique neuromolecular pathways linked specifically to suicide risk. These findings highlight important differences in gene regulatory mechanisms between MDD and MDD-associated suicide, potentially offering new insights into their pathophysiology.

The primary goal of this study was to investigate whether m6A RNA methylation contributes to the molecular pathology of MDD, and whether m6A methylation and associated gene functions differ between MDD subjects who died by suicide (MDD-S) and those who did not (MDD-NS). Our findings revealed significant m6A methylation changes in MDD compared to controls, with distinct gene methylation patterns in the MDD-NS vs. MDD-S groups. We also observed a significant inverse correlation between m6A methylation and gene expression. Functionally, abnormal m6A methylation disrupted the gene regulatory framework, impacting pathways related to neuronal integrity, synaptic function, neuroimmune activation, neurotransmission, and stress adaptation, which may increase the risk of suicide among MDD.

Our m6A methylation analysis in the dlPFC revealed a significant shift in methylation among MDD subjects compared to control subjects. We focused primarily on hypermethylated transcripts, as they play a role in suppressing gene expression. We found 1290 hypermethylated RNA transcripts in MDD, with notable clusters on chromosomes 9, 13, 16, and 22. The reason for this localized clustering remains unclear, however, it has been shown that these chromosomes are enriched with genes associated with neuronal function and development [23]. Another observation was that the majority of the methylation sites were significantly enriched in the coding regions, particularly within the exonic regions of mRNA transcripts, suggesting that m6A methylation may play a role in destabilizing protein-coding genes in the MDD group [24]. Additionally, our in-silico motif analysis revealed systematic mapping of the specific sequence motifs AAGA and ACCCA across m6A hypermethylated enrichment, indicating that these two motifs may serve as regulatory elements for m6A methylation in MDD subjects. The RRACH consensus sequence, known for its association with m6A RNA methylation, plays a crucial role in regulating gene expression. The MDD-enriched motifs AAGA and ACCCA align with this consensus sequence. Specifically, AAGA matches the RR and H positions of the motif, while ACCCA fits the purine and H positions [25]. Our findings indicate that AAGA and ACCCA motifs are likely crucial sites for m6A modification, influencing gene regulation and contributing to the pathophysiology of MDD and suicide risk.

Next, we examined the transcript levels of key m6A regulatory enzymes that are not only crucial for RNA methylation [26], but their role in cortical development, learning, cognitive function, and stress response has been previously demonstrated [27]. We observed that the expression of FTO, a demethylase that removes m6A marks, was significantly reduced in the MDD group. Conversely, METTL3, an m6A methyltransferase that adds m6A marks, exhibited increased expression, whereas no changes were noted for the methyltransferase METTL14. These results suggest a potentially compensatory relationship between methylation erasers and writers in MDD.

M6A methylation is a dynamic process that either enhances or suppresses gene expression [28]. Our identification of significantly hypermethylated genes in the MDD brain, particularly those involved in synaptic regulation and neuronal signaling, suggests that m6A methylation plays a critical role in the pathophysiology of MDD [16]. In the MDD group, M6A hypermethylated transcripts, particularly those involved in synaptic plasticity and neuronal connectivity, show an inverse correlation with their corresponding gene expression. Alterations in genes such as RAB5B, SRPX2, and TIAM1, which are involved in synaptic trafficking and cytoskeletal regulation, could further exacerbate synaptic dysfunction in MDD [29]. This finding not only highlights the regulatory role of m6A in shaping the transcriptomic landscape of the MDD brain [30] but also points to potential disruptions in neuronal communication and synaptic function in MDD [31].

The results from our significantly hypermethylated gene function network (connectivity map) offer a novel perspective on the role of m6A RNA methylation-based epitranscriptomic regulation in the complex gene regulation processes underlying the MDD brain. Identifying hub-methylated transcripts as central drivers in the network connectivity map suggests that m6A modifications may be pivotal in orchestrating the functional connectivity of genes involved in cellular processes critical to mood regulation and neuroplasticity. The identification of methylation hotspots within key genes linked to neurotransmitter signaling and cellular stress responses suggests that m6A RNA methylation plays a crucial role in modulating pathways such as cAMP, dopaminergic, and GABAergic signaling. This further reinforces the connection between m6A-regulated genes and dysregulated signaling pathways known to be implicated in MDD. This underscores the potential of m6A RNA methylation in influencing gene expression and neuronal function, contributing to MDD pathophysiology.

Our gene function analysis of methylated transcripts offers further insights into the functional consequences of m6A methylation changes in MDD. GO network analysis of MDD-associated differentially methylated transcripts indicated substantial enrichment in synaptic functions, including synaptic vesicle trafficking, presynaptic assembly, and postsynaptic organization. Hypermethylated genes in the MDD group, such as RAB5B, EFNB1, SDCBP, and SNAP47, have been shown to serve as key regulators of synaptic vesicle mobilization and neurotransmitter release [32]. RAB5B plays a crucial role in early endosome trafficking and is linked to synaptic vesicle recycling, a process essential for sustained neurotransmitter release and synaptic plasticity [33]. EFNB1 and SDCBP are involved in synaptic development and neuronal connectivity; their altered expression may contribute to synaptic destabilization and impaired plasticity [34], phenomena often observed in MDD [31]. SNAP47, a SNARE-associated protein, regulates synaptic vesicle exocytosis, and its hypermethylation could lead to dysfunctional neurotransmitter release and impaired synaptic efficacy [35]. Further investigation into neuronal structure-related GO terms for identified hypermethylated genes CAMK2A, SHANK3, and DLG4 (PSD-95) involved in dendritic spine morphogenesis and synaptic plasticity suggest impaired synaptic architecture and reduced synaptic strength, critical in MDD pathology [36, 37]. CAMK2A is a key kinase in long-term potentiation, playing an important role in synaptic strengthening and memory formation [38]. Its altered methylation status may lead to deficits in cognitive function and synaptic adaptability, hallmarks of MDD [39]. The role of SHANK3, as a scaffolding protein, in maintaining synaptic integrity may link its dysregulation to disrupted excitatory synapse function, contributing to the reduced synaptic density observed in MDD patients [40]. Similarly, DLG4 (PSD-95), a key regulator of postsynaptic receptor clustering and synaptic stability, is critical for maintaining glutamatergic synapses [41]. Hypermethylation of DLG4 may impair excitatory synaptic transmission, thereby contributing to the synaptic dysfunction observed in MDD [42]. In parallel, GO terms related to stress response and neuroimmune pathways were significantly enriched in MDD, with hypermethylation identified in key immune-regulatory genes such as IL6, TNF-α, NF-κB, and NLRP3. These findings align well with previous studies suggesting that chronic inflammation and dysregulated immune signaling are key contributors to MDD pathophysiology [43–49]. The hypermethylation of these immune-related genes further indicates a maladaptive response that could contribute to neuronal stress, deficits in synaptic remodeling, and, ultimately, the persistence of depressive symptoms [50].

In the MDD group, 17 out of 49 subjects died by suicide (MDD-S), while 32 died from causes other than suicide (MDD-NS). We examined whether the m6A methylation alterations were comparable or dissimilar across these two populations. Our comparison analysis showed that 196 m6A hypermethylated genes were specifically associated with suicide among the MDD population [11]. GO analysis in the MDD-S group exhibited unique methylation patterns affecting pathways associated with neuronal development, neuroinflammation, and neurotransmitter signaling. Differential methylation in genes implicated in axon guidance (SEMA5A, ROBO2, SHH), MAPK signaling (PIK3CA, CAMK2B), and synaptic function suggests a profound impact on neuronal connectivity and signal transduction, potentially increasing vulnerability to suicide [51]. SEMA5A and ROBO2 are crucial for axonal pathfinding and synaptic organization [52]. SHH, an essential regulator of neurogenesis and axonal targeting, has been implicated in mood disorders and could contribute to impaired neural plasticity in suicidal individuals [53]. Additionally, neurotransmitter-associated pathways, particularly glutamatergic and dopaminergic signaling, showed significant enrichment of hypermethylated genes, including GRIK4, GRM1, CACNA1C, and ADCY2. These alterations may affect excitatory neurotransmission, synaptic efficiency, and neuronal excitability [54–56]. GRIK4 encodes a kainate receptor subunit involved in glutamatergic synaptic transmission, and its dysregulation is linked to major psychiatric disorders [57]. GRM1, a metabotropic glutamate receptor, plays a role in modulating synaptic strength, and its epigenetic alteration may contribute to imbalanced excitatory-inhibitory neurotransmission [58]. CACNA1C, a gene that encodes the L-type voltage-gated calcium channel, has been repeatedly associated with mood disorders, impacting neuronal excitability and plasticity [59, 60]. ADCY2, which regulates the synthesis of cyclic AMP and dopamine signaling, has been linked to suicidal behavior, further supporting its involvement in affective dysregulation [61]. Further analysis of cellular components in the MDD-S group revealed significant m6A enrichment in transcripts related to postsynaptic density, mitochondrial function, and endoplasmic reticulum-associated processes, indicating widespread disruptions in synaptic plasticity and cellular metabolism [62]. Notably, changes in the methylation of mitochondrial and ER-associated genes may reflect dysregulated energy homeostasis and stress response mechanisms, which have been implicated in suicide pathophysiology [63, 64]. PPi network analysis underscored the importance of stress response and neurotrophic signaling, highlighting interactions involving corticotropin-releasing factor and brain-derived neurotrophic factor (BDNF) [65, 66]. BDNF is crucial for synaptic plasticity and neuronal survival, and its decreased expression has been consistently linked to depression and suicidal behavior [64, 67]. GABRB3, RAF1, and TNF exhibited significant methylation alterations, indicating potential impairments in GABAergic neurotransmission and neuroimmune interactions, which may compromise stress adaptation mechanisms [43, 68, 69]. Additionally, differential methylation in calcium-signaling genes (CACNA1A, CAMK2G, PRKCA) suggests a broader impact on synaptic activity and neuronal plasticity, supporting the hypothesis that m6A dysregulation contributes to impaired neural circuits underlying suicide risk [70].

The MDD-S group was comprised of 9 suicide subjects who died by violent means and 8 by non-violent means. Suicide by violent means is considered a distinct subgroup within the suicide completers, differing not only in method but also in clinical, neurobiological, and genetic characteristics [71]. Our differential m6A methylation analysis provides novel insights into the role of m6A RNA methylation in modulating suicide pathophysiology, particularly in distinguishing violent from non-violent suicide completers within a cohort of individuals diagnosed with MDD. The identification of 31 significantly hypermethylated and 131 hypomethylated gene transcripts in violent suicide completers underscores the existence of a specific m6A epitranscriptomic landscape associated with the violent method of suicide. The identification of a unique 20 hypermethylated gene transcripts further suggests a divergent molecular pathway contributing to lethality or the mode of suicide method. Altogether, our study suggests that violent suicide is not just a behavioral outcome but also associated with underlying molecular differences at the post-transcriptional regulatory level.

Our study has some limitations. First, whereas we used a custom modification made microarray, which was highly effective in detecting m6A modifications at known conserved sites across the genome, further study using MeRIP-sequencing, which provides genome-wide coverage, should be pursued. Second, we examined the transcripts of m6A readers, writers, and erasers using qPCR. It would be interesting to see if similar changes occur at the protein level. Also, the sample size, especially for the MDD-S group, was relatively small. While our findings suggest significant differences in m6A methylation, future research involving larger cohorts should validate these results. Lastly, our study focused on the dorsolateral prefrontal cortex. Although this brain area plays an essential role in mood regulation, other regions such as the hippocampus and amygdala are also critically important in the pathophysiology of MDD and suicide. Future studies should investigate m6A methylation changes in these brain areas for a comprehensive understanding of the neurobiological mechanisms at play.

Altogether, our findings indicate that m6A RNA methylation is a crucial epitranscriptomic mechanism underlying the neurobiology of MDD. The unique m6A methylation profiles identified in this study offer valuable insights into the molecular processes contributing to MDD pathology and the heightened risk of suicide. While m6A methylation is involved in key brain processes such as synaptic signaling, stress responses, and neuroinflammation, further research is required to track how these changes evolve over time and relate to suicidal behavior. Studies that integrate genetic, protein, and metabolic data will be essential to unravel the complex connections between m6A methylation and suicide risk. Our findings also suggest that m6A methylation could be a potential biomarker for identifying individuals at higher risk for suicide in the context of depression. Understanding how m6A regulates critical brain functions opens the door for targeted therapies that could modify m6A methylation to prevent suicide. This could ultimately lead to novel treatment strategies aimed at managing depression and reducing suicide risk by altering m6A-mediated gene regulation.

## Supplementary information

Supplementary section

Table S3

Table S4

Table S5

Table S8

Table S10

Table 11

Table S12

Table S13

Table S15

Table S16

Table S14

## Data Availability

All data generated or analyzed during this study are included in this published article [and its supplementary information files].
