# Supplementary material for: M6A RNA epitranscriptome dynamics linked to major depressive disorder and suicide risk
Source: Neuropsychopharmacology. 2025 Jul 10;50(10):1524–35. doi: 10.1038/s41386-025-02165-5 (PMC12340114; doi:10.1038/s41386-025-02165-5)
Supplement: Supplementary file 1 — Supplementary section [file 41386_2025_2165_MOESM1_ESM.docx]

**SUPPLEMENTARY SECTION**

**M^6^A RNA Epitranscriptome Dynamics Linked to Major Depressive Disorder and Suicide Risk**

Bhaskar Roy, Ph.D. and Yogesh Dwivedi, Ph.D.

Department of Psychiatry and Behavioral Neurobiology

Heersink School of Medicine

University of Alabama at Birmingham, Birmingham, Alabama, USA 35242

**^*^Corresponding author:**

Yogesh Dwivedi, Ph.D.

Distinguished Professor of Psychiatry

Elesabeth Ridgely Shook Endowed Chair for Psychiatric Research

Director of Translational Research, UAB Mood Disorder Program

Co-Director, UAB Depression and Suicide Center

Department of Psychiatry and Behavioral Neurobiology

University of Alabama at Birmingham

SC711 Sparks Center

1720 7^th^ Avenue South

Birmingham, Alabama, USA

Phone: 1-205-975-8459

Email: [yogeshdwivedi@uabmc.edu](mailto:ydwivedi@uabmc.edu)

**SUPPLEMENTAL METHODS**

**Subjects**

The study was approved by the Institutional Review Board of the University of Alabama at Birmingham. Samples from dlPFC were obtained from the Maryland Brain Collection. The cohort comprised 98 brain samples, among which 17 were MDD subjects who died by suicide (MDD-S), 32 were MDD subjects who died by causes other than suicide (MDD-NS), and 49 were control subjects without psychiatric disorders (referred to as controls). Tissues were collected only after a family member gave informed consent.  All tissues from controls and MDD subjects were screened for evidence of neuropathology and were excluded if they exhibited features of Alzheimer’s disease, infarctions, demyelinating diseases, or atrophy (or clinical history of these disorders). Toxicology and the presence of antidepressants were examined by analysis of urine and blood samples from these subjects. Brain pH was measured as described previously.^1^ Detailed demographic and clinical characteristics of subjects are shown in **Table S1**. The psychiatric diagnosis was determined by psychological autopsy as described earlier^2^ using Diagnostic Evaluation After Death (DEAD)^3^ and the Structured Clinical Interview for the DSM-5 (SCID)^4^. The interviews were done by a trained psychiatric social worker. Two psychiatrists independently reviewed the write-up from this interview, as well as the SCID that was completed from it, as part of their diagnostic assessment of the case. Diagnoses were made from the data obtained in this interview, medical records from the case, and records from the Medical Examiner's office. The two diagnoses were compared, and discrepancies were resolved by means of a consensus conference. Control subjects were verified as free from mental illnesses using these consensus diagnostic procedures.

After removal from the cranium, the brains were cut into six major pieces (four cerebral cortical lobes, basal ganglia-diencephalon, and lower brain stem-cerebellum), rapidly frozen on dry ice, and stored at −70°C until dissection. During dissection, the frontal lobes were sliced into 1-mm to 1.5-mm thick coronal sections at a temperature between 0°C and 10°C. To keep the samples frozen, the dissections were performed on a metal plate over a container filled with dry ice. The prefrontal cortical samples were cut out of the coronal sections by a fine microdissecting (Graefe) knife under a stereomicroscope with low magnification. The dorsomedial prefrontal cortex (Brodmann's area 9) was taken just dorsal to the frontopolar area, including the most polar portion of the superior and partly the middle frontal gyrus between the superior and intermediate frontal sulci. In the sections of the dissected cortical area, the gray and white matter were separated. The tissues were chopped into smaller pieces and stored at −80°C until use.

All tissues from the control and suicide groups were screened for evidence of neuropathology by experienced neuropathologists at each brain collection program. The tissues were examined histologically. Fixed sections of PFC were screened with hematoxylin and eosin (H&E) staining and an antibody to glial fibrillary acid protein. Alzheimer’s disease, infarcts, demyelinating diseases, or atrophy disqualified subjects from the study. In addition, in each case, screening for the presence of human immunodeficiency virus (HIV) was done in blood samples, and all HIV-positive cases were excluded. Toxicology data were obtained by the analysis of urine and blood samples.

### **RNA Isolation and m^6^A Methylation Enrichment Analysis Following m^6^A mRNA Immunoprecipitation (MeRIP)**

To map the m6A methylome at high resolution, dlPFC (~50 mg tissue) was used to isolate total RNA following the Trizol (Invitrogen, Life Technologies)- based method. However, to find out the nucleus-specific m6A methylation status, nuclei from cortical tissue slices were enriched to perform RNA extraction. Briefly, the cell pellet was incubated in five volumes of isotonic lysis buffer (ILB; 10 mM Tris-HCl pH 7.5, 3 mM CaCl2, 2 mM MgCl2, 0.32 M sucrose, 1,000 U/ml RNAsin RNase inhibitor (Promega, USA), 1 mM DTT) for 10 min to induce cell swelling. Then, Triton X-100 was added to a final concentration of 0.3%, and cells were incubated for 6 min to lyse the plasma membranes. Nuclei were pelleted at 600g for 5 min and washed once with ten volumes of ILB. RNA was extracted from the nuclear pellet using Trizol (Invitrogen, Life Technologies, USA). Residual contaminating DNA was digested using the RNase-free DNase set from Qiagen (USA). RNA was re-purified by sequential acid phenol-chloroform and chloroform extractions followed by ethanol precipitation. RNA integrity and absence of DNA were confirmed by Bioanalyzer RNA Nano chips (Agilent Technologies, USA). Samples with RIN>7 were used for further analysis.

For RNA immunoprecipitation, 200 nt long random RNA fragments were produced from 1 µg of total RNA. RNA fragmentation was performed in the presence of ZnCl_2_ salt using 1X RNA fragmentation buffer (10 mM Tris-HCl [pH 7.0] and 10 mM ZnCl_2_). The incubation time in the fragmentation step was adjusted to determine the length of RNA fragments. Fragmented RNA was precipitated with ethanol to minimize the possible carryover of ZnCl_2_ to the next step. Meanwhile, a total amount of 500 ng fragmented RNA was denatured at 75°C for 5 minutes. Next, the denatured-fragmented RNA was diluted in 250 µl of IP buffer (150 mM NaCl, 0.1% NP-40, 10 mM Tris-HCl, pH 7.4). A fraction of fragmented RNA (10% of the amount used for MeRIP) was set aside as pre-MeRIP input control. For MeRIP, 3 μg of RNA and m6A spike-in control mixture were added to the IP buffer (50 mM Tris-HCl, pH 7.4, 150 mM NaCl, 0.1% NP40, 40 U/μL RNase inhibitor) and incubated with 2 μg anti-m6A rabbit polyclonal antibody (cat#202003, Synaptic Systems, Germany) at 4°C for 2h. Dynabeads™ M-280 sheep anti-rabbit IgG (cat#11203D, Invitrogen, USA) were blocked with 0.5% BSA at four °C for 2h, washed, resuspended in the total RNA-antibody mixture prepared above, and incubated at 4°C for a further 2h. The modified RNA was eluted with elution buffer (10 mM Tris-HCl, pH 7.4, 1 mM EDTA, 0.05% SDS, 40 U Proteinase K) at 50°C for 1h, and the unmodified RNA was recovered from the supernatant. The modified RNA and unmodified RNA were extracted by acid phenol-chloroform and ethanol precipitation, respectively.

**Microarray Hybridization and Analysis**

**RNA Isolation and Labelling**

Modified RNAs were extracted from the immunoprecipitated magnetic beads and labeled as “IP,” while unmodified RNAs were recovered from the supernatant and labeled as “Sup.” The “IP” and “Sup” RNAs were subsequently labeled with Cy5 and Cy3, respectively, as cRNAs, following the Arraystar RNA Labeling protocol (Arraystar Inc., USA). The cRNAs were combined and hybridized onto the Arraystar Human 8x60K mRNA Epitranscriptomic Microarray (Arraystar Inc., USA). Following hybridization, the slides were washed and scanned in two-color channels using an Agilent Scanner G2505C (Agilent, USA). Calibration spike-in control RNA was added to both the “IP” and “Sup” RNA samples in equal amounts, with the “Sup” RNA labeled with Cy3 and the “IP” RNA labeled with Cy5. This labeling procedure was carried out using the Arraystar Super RNA Labeling Kit (Arraystar Inc., USA). The synthesized cRNAs were purified using the RNeasy Mini Kit, and their concentration and specific activity (pmol dye/μg cRNA) were measured with the NanoDrop ND-1000.

**RNA Fragmentation and Hybridization**

2.5 μg of both Cy3- and Cy5-labeled cRNAs were mixed, and the cRNA mixture was fragmented by adding 5 μL of 10X Blocking Agent and 1 μL of 25X Fragmentation Buffer. The reaction was heated at 60°C for 30 minutes and then combined with 25 μL of 2X Hybridization Buffer. A total of 50 μL of the hybridization solution was dispensed onto a gasket slide and assembled onto the m6A-mRNA Epitranscriptomic Microarray slide, containing 44,122 mRNAs. The slides were incubated at 65°C for 17 hours in an Agilent Hybridization Oven. Following the hybridization step, the arrays were washed, fixed, and scanned using the Agilent Scanner G2505C.

**Data Analysis**

The acquired array images were analyzed using the Agilent Feature Extraction software (version 11.0.1.1). Raw intensities for the “IP” (Cy5-labeled) and “Sup” (Cy3-labeled) samples were normalized with the average log2-scaled intensities of the spike-in RNA controls. The m6A methylation level was calculated based on the normalized Cy5-labeled “IP” intensities. The raw intensities of Cy5-labeled and Cy3-labeled RNAs were normalized with the average log2-scaled spike-in RNA control intensities. The m6A methylation percentage was calculated based on the normalized intensities of the Cy5- and Cy3-labeled RNAs. Fold change and p-values were determined for each transcript between the comparison groups. Differentially m6A-methylated RNAs were identified by applying a fold change (FC) cutoff of ≥1.5 or ≤0.7 and a p-value threshold of <0.05. The differential changes were then adjusted for multiple testing using the Benjamini-Hochberg false discovery rate (FDR) correction method.^5^ Lastly, hierarchical clustering was performed to visualize the differential m6A-methylation patterns among the samples.

**Isolation of RNA and Bulk RNA Sequencing**

**RNA Isolation**

TRIzol ® (Invitrogen, USA) was used to isolate RNA from total dlPFC homogenates following the method described earlier.^6^ RNA purity was checked by Nanodrop (260/280 nm; cutoff ≥1.8) and their integrity by agarose gel electrophoresis.

**Construction and Sequencing of cDNA Library**

Approximately 1-2 µg total RNA of each sample was used for RNA-seq library preparation. Briefly, mRNA was isolated from total RNA with NEBNext® Poly(A) mRNA magnetic isolation module (New England Biolabs, USA). rRNA was removed from total RNA with a RiboZero magnetic gold kit (Epicenter, USA). The enriched mRNA or rRNA depleted RNA was used for RNA-seq library preparation using KAPA stranded RNA-Seq library prep kit (Illumina, USA). The library preparation procedure included: 1) fragmentation of RNA molecules; 2) reverse transcription (RT) to synthesize first strand cDNA; 3) second-strand cDNA synthesis incorporating dUTP; 4) end-repair and A-tailing of the double-stranded cDNA; 5) Illumina compatible adapter ligation; and 6) PCR amplification and purification. The completed libraries were qualified on Agilent 2100 Bioanalyzer for concentration, fragment size distribution (400-600 bp), and adapter dimer contamination. The amount was determined by the absolute quantification qPCR method. The barcoded libraries were mixed in equal amounts and used for sequencing. The DNA fragments in well-mixed libraries were denatured with 0.1M NaOH to generate single-stranded DNA molecules, loaded onto channels of the flow cell at 8 pM concentration, and amplified in-situ using TruSeq SR Cluster Kit v3-cBot-HS (Illumina, USA). Sequencing was carried out by running 150 cycles using Illumina HiSeq4000, according to the manufacturer’s instructions.

**Bio-computational Analysis of RNA Sequencing Data**

Raw data files in FASTQ format were generated from the Illumina sequencer. To examine the sequencing quality, the quality score plot of each sample was plotted and examined using the FastQC software. After quality control, the fragments were 5’, 3’-adaptor trimmed and filtered ≤20 bp reads with cutadapt software. The trimmed reads were aligned to reference genome with Hisat 2 software. Based on alignment statistical analysis (mapping ratio, rRNA/mtRNA content, fragment sequence bias), results were used for subsequent data analysis. The expression levels (FPKM value) of known genes and transcripts were calculated using ballgown through the transcript abundances estimated with StringTie. The number of identified genes/groups was calculated based on the mean FPKM (P≥0.5). Heatmap and k-means clustering were visualized for the expressed genes using the iDEP tool suit 25. Differentially expressed gene analysis was performed with the R package ballgown. Expressed genes were used to create Volcano plots using R (v.3.6.3) library. The threshold for the p-value cutoff of the expressed gene was assigned ≤0.05. Up- and down-regulated genes are depicted by red and green color dots, respectively. The remaining insignificant genes are depicted as dark gray dots. The x- and y-axes correspond to the log2 fold change value and the mean expression value of log 10 (p-value) respectively.

**cDNA Synthesis and Quantitative PCR (qPCR) to Analyze the Expression of M6A Modifying Enzymes**

The expression levels of various m6A methylating/demethylating enzymes were determined by qPCR, as previously described^6^ and detailed in the following section. The forward and reverse primer sequences are provided in **Table S2**. For qPCR, 500ng RNA was used to synthesize cDNA using M-MLV Reverse Transcriptase (Invitrogen, USA) and oligo (dT) primer as previously described.^6^ The relative transcript expression was measured with 1X EvaGreen qPCR mastermix (Applied Biological Material Inc., Canada) and 0.8 μM of gene-specific forward and reverse primers (**Table S2**). Twenty-fold diluted cDNA was used as a template to conduct qPCR with the following protocol: initial denaturation at 95°C for 10 min, repeating 40 cycles of denaturation at 95°C for 10 sec, primer annealing at 60°C for 15 sec, and an elongation at 72°C for 20 sec. To exclude the possibility of primer dimer formation and secondary product amplification, EvaGreen-specific dissociation curve analysis was performed following an initial denaturation at 72°C for 1 min, annealing at 55°C for 30 sec, and a repeat denaturation step at 95°C for 30 sec. GAPDH was used as a housekeeping gene. Fold change was calculated following Livak’s ΔΔCt method.^6^

**Gene Ontology (GO) and Functional Clustering of M6A-Enriched Coding Transcripts**

GO term enrichment analyses were performed using David (http://david. abcc. ncifcrf.gov/).^7^ The analysis results from DAVID were further plugged into the ClueGO app (v2.5.3) within Cytoscape (v3.7.2), enabling GO Term Fusion to combine terms with very similar gene lists while using a custom background corresponding to expressed genes obtained from the input samples. The resulting enriched GO terms were visualized with a custom script using ggplot2 (v3.3.5), displaying the adjusted p-value for each GO term, the number of genes from the list that belong to said term, and the percentage of the total genes in the GO term that are present in the list. Synaptic GO enrichment analyses were performed with SynGO (v1.1, syngoportal.org). Only GO terms with P<0.05 were determined statistically significant. Plots were prepared using R Scripts on a standalone R Studio platform.

**RNA Expression vs. M6A Methylation Correlation Analysis**

Pearson correlation analysis was performed to assess the direction and strength of expression correlations between m6A methylation enrichment and mRNA expression data. Correlation coefficients (R) and statistical significance (p) values were calculated to assess the relationship between m6A-based mRNA methylation and gene expression. A correlation was considered noteworthy if it met the threshold of |R| ≥ 0.3 and approached statistical significance at p<0.05.

**Statistical Analysis**

Statistical analyses were conducted using SPSS (v.29, IBM, USA). The Shapiro-Wilk test was used to assess the normality of the data. The average difference of age, PMI, and brain PH was assessed by the student’s t-test. Differences in gender, drug abuse, alcohol abuse, and antidepressant toxicology were analyzed by Fisher's exact test. The average difference in gene expression was compared by the student’s t-test. The correlation between the fold change of RNA-seq and qPCR was calculated with the Pearson correlation coefficient. Correlations of the gene expressions with covariates were also conducted with the Pearson correlation coefficient. Statistical significance was set at the 95% level (p≤0.05).

| **Table S1: Demographic characteristics of subjects** | | | | |
| --- | --- | --- | --- | --- |
|  | **Non-psychiatric Controls (C)**  **(N=49)** | **Total MDD**  **(MDD-S/MDD-NS)**  **(N=49)** | **MDD-NS**  **(N=32)** | **MDD-S**  **(N=17)** |
| Age (Year) | 47.75±2.48 | 43.71±2.33  (F=0.24, t=1.18, df=96, p=0.24) | 38.96±2.95  (p=0.15) | 50.66±4.01  (p=0.15)  (MDD-S vs MDD-NS: p=0.16) |
| PMI (Hours) | 18.14±0.88 | 21.53±2.43  (F=1.13, t=-1.31, df=96, p=0.19) | 23.17±3.91  (p=0.25) | 17.93±2.25  (p=1.0)  (MDD-S vs MDD-NS: p=0.62) |
| Brain pH | 7.10+0.03 | 7.08±0.03  (F=0.94, t=0.52, df=96, p=0.61) | 7.04±0.03  (p=1.0) | 7.09±0.05  (p=1.0)  (MDD-S vs MDD-NS: p=0.90) |
| RIN | 7.66±0.04 | 7.68±0.04  (F=0.002, t=-0.26, df=96, p=0.39) | 7.64±0.04  (p=1.0) | 7.59±0.08  (p=1.0)  (MDD-S vs MDD-NS: p=0.08) |
| Sex  Males  Females | 29  20 | 30  19 | 19  13 | 11  6 |
| Cause of Death | Lymphoma, PE, cardiac failure, lung cancer, pneumonia, ACI, ASCVD, CA, MVA, colon cancer, MI, leukemia, hemopericardium, LS, GSW, electrocution, hemopericardium,  morbid obesity, drowning | GSW, jumped from height, hanging, CO intoxication, drug overdose, stab wound, ASCVD, MVA, ketoacidosis, cardiomegaly, seizure, fatty liver | ASCVD, MVA, ketoacidosis, cardiomegaly, seizure, fatty liver | GSW, jumped from height, hanging, CO intoxication, drug overdose, stab wound |
| Race | 8 Black/41 White | 7 Black/41 White/1 Asian | 3 Black/1 Asian/28 White | 4 Black/13 White |
| Neurological/  Neuropathological disorders | None | None | None | None |
| Number of subjects with positive antidepressant toxicology | None | 19 | 12 | 7 |
| Number of subjects with substance use | None | 4 | 1 | 3 |
| Number of subjects with positive alcohol toxicity | None | 18 | 13 | 5 |
| ACF=acute cardiac failure; ACI=accidental chest injury; AMI=acute myocardial infarction; ASCVD=atherosclerotic cardiovascular disease; CA=cardiac arrhythmia; GSW=gunshot wound; LS=lung sarcoidosis; MVA=multiple vehicle accident; MVP=mitral valve prolapse; PM=pulmonary embolism; MDD=major depressive disorder; MDD-S=MDD subjects who died by suicide; MDD-NS=MDD subjects who died by causes other than suicide. Data are the mean ± SEM. The data were analyzed using independent sample t-test. MDD-S and MDD-NS groups were compared with the control group using On-way ANOVA followed by Bonferroni correction. Overall ANOVA between the three groups of subjects were as follows: Age: F=2.64, df=2,95, p=0.08; PMI: F=1.67, df=2,95, p=0.19; brain pH: F=0.68, df=2,95, p=0.51; and RIN: F=1.23, df=2,95, p=0.29. In 7 MDD cases, alcohol toxicity was not determined. | | | | |

| **Table S2. Primer sequences used in qPCR assays** | | |
| --- | --- | --- |
| **Oligo ID** | **Sequence (5'-3')** | **Assay Type** |
| hsa-Fto Forward | GCATGACCCAGCCTATGGTT | Gene expression |
| hsa-Fto Reverse | GGTGATGCAGGTCAGGTTCA | Gene expression |
| hsa-Mettl3 Forward | ACAACAGCCAAGGAACAATCC | Gene expression |
| hsa-Mettl3 Reverse | GCACTCCTCCTTGGTTCCATA | Gene expression |
| hsa-Mettl14 Forward | CACTTCCAGCTTGCACTTTGC | Gene expression |
| hsa-Mettl14 Reverse | GACAACTGCAAGCAAGGCTC | Gene expression |
| hsa-Gapdh Forward | CCACATCGCTGAGACACCAT | Gene expression |
| hsa-Gapdh Reverse | AGTTAAAAGCAGCCCTGGTGA | Gene expression |

| **Table S6: Significantly M6A hypermethylated gene transcripts relevant to CNS function** | | |  |
| --- | --- | --- | --- |
|  |  |  |  |
| **Gene Symbol** | **m6A Fold-change** | **m6A P-value** |  |
| RAB5B | 2.30576306 | 0.023198049 |  |
| EFNB1 | 1.73316424 | 0.035236245 |  |
| SDCBP | 2.04639489 | 0.006747105 |  |
| SNAP47 | 1.62756504 | 0.012529452 |  |
| SLC3A2 | 1.6687028 | 0.000980731 |  |
| PLAT | 1.62507253 | 0.048724658 |  |
| TIAM1 | 2.01845252 | 0.041079547 |  |
| NR3C1 | 1.84632133 | 0.047955361 |  |
| NUMB | 1.61364592 | 0.020945443 |  |
| LZTS1 | 1.83958941 | 0.007051797 |  |
| CHD4 | 1.50578754 | 0.028517788 |  |
| EIF3L | 3.66563063 | 0.01390525 |  |
| HNRNPL | 1.74661273 | 0.018711976 |  |
| ATP6V0A1 | 2.01030858 | 0.016242905 |  |
| PTCH1 | 1.88891975 | 0.000899069 |  |
| SAMD4A | 2.50690737 | 0.009172356 |  |
| DCX | 2.66658871 | 0.003164395 |  |
| SRPX2 | 2.3015181 | 0.042235551 |  |
| DMTN | 2.18013219 | 0.007373023 |  |
| KIF3A | 1.61736776 | 0.022055231 |  |

| **Table S7: Transcripts specific m6A methylation vs expression changes in the MDD group** | | | | |
| --- | --- | --- | --- | --- |
| **Gene** | **m6A Fold-change** | **m6A P-value** | **Exprsn Fold-change** | **P-value** |
| TIAM1 | 2.018452517 | 0.041079547 | -1.180559143 | 0.417145147 |
| DCX | 2.666588709 | 0.003164395 | -1.203006334 | 0.589316817 |
| SNAP47 | 1.627565038 | 0.012529452 | -1.204181424 | 0.535479473 |
| SRPX2 | 2.301518103 | 0.042235551 | -1.24110912 | 0.829324259 |
| RAB5B | 2.305763057 | 0.023198049 | -1.496974105 | 0.073455085 |
| PTCH1 | 1.888919749 | 0.000899069 | -1.611746173 | 0.109652837 |
| ATP6V0A1 | 2.010308577 | 0.016242905 | -1.653621255 | 0.009305332 |
| NUMB | 1.613645916 | 0.020945443 | -2.091777671 | 0.018457733 |
| NR3C1 | 1.846321325 | 0.047955361 | -2.171235966 | 0.002940205 |
| EIF3L | 3.665630629 | 0.01390525 | -2.39854219 | 0.002566619 |
| KIF3A | 1.617367762 | 0.022055231 | -2.455614748 | 1.76488E-05 |
| SDCBP | 2.046394888 | 0.006747105 | -6.187110374 | 2.1191E-09 |

| **Table S9: Gene ontology terms enriched for synaptic functions based on significantly hypermethylated transcripts in the MDD group** | | | |
| --- | --- | --- | --- |
| **Gene Symbol** | **GO Term ID** | **GO Term Name** | **GO Domain** |
| RAB5B | GO:0098993 | anchored component of synaptic vesicle membrane (GO:0098993) | CC |
| EFNB1 | GO:0099056 | integral component of presynaptic membrane (GO:0099056) | CC |
| EFNB1 | GO:0099054 | presynapse assembly (GO:0099054) | BP |
| SDCBP | GO:0099054 | presynapse assembly (GO:0099054) | BP |
| SNAP47 | GO:0048786 | presynaptic active zone (GO:0048786) | CC |
| SLC3A2 | GO:0045202 | synapse (GO:0045202) | CC |
| SNAP47 | GO:0014069 | postsynaptic density (GO:0014069) | CC |
| PLAT | GO:0098794 | postsynapse (GO:0098794) | CC |
| PLAT | GO:0099183 | trans-synaptic signaling by BDNF, modulating synaptic transmission (GO:0099183) | BP |
| PLAT | GO:0098992 | neuronal dense core vesicle (GO:0098992) | CC |
| PLAT | GO:0099544 | extrasynaptic space (GO:0099544) | CC |
| PLAT | GO:0098794 | postsynapse (GO:0098794) | CC |
| TIAM1 | GO:1905274 | regulation of modification of postsynaptic actin cytoskeleton (GO:1905274) | BP |
| TIAM1 | GO:0099147 | extrinsic component of postsynaptic density membrane (GO:0099147) | CC |
| SNAP47 | GO:0098967 | exocytic insertion of neurotransmitter receptor to postsynaptic membrane (GO:0098967) | BP |
| NR3C1 | GO:0099092 | postsynaptic density, intracellular component (GO:0099092) | CC |
| NUMB | GO:0014069 | postsynaptic density (GO:0014069) | CC |
| NUMB | GO:0099149 | regulation of postsynaptic neurotransmitter receptor endocytosis (GO:0099149) | BP |
| NUMB | GO:0150052 | regulation of postsynapse assembly (GO:0150052) | BP |
| LZTS1 | GO:0099031 | anchored component of postsynaptic density membrane (GO:0099031) | CC |
| LZTS1 | GO:0150052 | regulation of postsynapse assembly (GO:0150052) | BP |
| TIAM1 | GO:0099175 | regulation of postsynapse organization (GO:0099175) | BP |
| CHD4 | GO:0051963 | regulation of synapse assembly (GO:0051963) | BP |
| EIF3L | GO:0045202 | synapse (GO:0045202) | CC |
| HNRNPL | GO:0045202 | synapse (GO:0045202) | CC |
| RAB5B | GO:0099525 | presynaptic dense core vesicle exocytosis (GO:0099525) | BP |
| SDCBP | GO:0098793 | presynapse (GO:0098793) | CC |
| ATP6V0A1 | GO:0097401 | synaptic vesicle proton loading (GO:0097401) | BP |
| ATP6V0A1 | GO:0030285 | integral component of synaptic vesicle membrane (GO:0030285) | CC |
| PTCH1 | GO:0099055 | integral component of postsynaptic membrane (GO:0099055) | CC |
| SAMD4A | GO:0014069 | postsynaptic density (GO:0014069) | CC |
| SAMD4A | GO:0150052 | regulation of postsynapse assembly (GO:0150052) | BP |
| DCX | GO:0098793 | presynapse (GO:0098793) | CC |
| DCX | GO:0150052 | regulation of postsynapse assembly (GO:0150052) | BP |
| SRPX2 | GO:0045202 | synapse (GO:0045202) | CC |
| SRPX2 | GO:0051963 | regulation of synapse assembly (GO:0051963) | BP |
| DMTN | GO:0045202 | synapse (GO:0045202) | CC |
| KIF3A | GO:0098937 | anterograde dendritic transport (GO:0098937) | BP |
| KIF3A | GO:0098794 | postsynapse (GO:0098794) | CC |
| KIF3A | GO:0098971 | anterograde dendritic transport of neurotransmitter receptor complex (GO:0098971) | BP |

| **Table S16: Uniquely hypermethylated gene transcripts in violent suicide completers** | | |
| --- | --- | --- |
| **GeneSymbol** | **Fold-change** | **log2-FC** |
| DTNB | 1.250349508 | 0.322331426 |
| ZSCAN26 | 1.324768206 | 0.405739954 |
| DACH2 | 1.333282196 | 0.414982166 |
| TPD52L1 | 1.343376815 | 0.425864035 |
| PHRF1 | 1.380313289 | 0.464995752 |
| ZNF615 | 1.385949326 | 0.47087451 |
| FLT1 | 1.443075099 | 0.529146381 |
| ZNF248 | 1.489382812 | 0.574714613 |
| ITPR3 | 1.539817069 | 0.622758968 |
| ESCO1 | 1.550194376 | 0.632449124 |
| NFYA | 1.558649815 | 0.640296831 |
| PKD1L3 | 1.690449524 | 0.757406939 |
| PKD1L3 | 1.730357433 | 0.791070081 |
| DYSF | 1.778629808 | 0.830766269 |
| NAA15 | 1.796543678 | 0.845224011 |
| RPL35 | 2.148406615 | 1.103267069 |
| GRP | 2.187942889 | 1.129575081 |
| BAZ1B | 2.241815323 | 1.164667436 |
| DIAPH1 | 2.329375889 | 1.219943465 |
| SAMD12 | 2.629445591 | 1.394758644 |

**Figure S1:** Effect of age, PMI, and brain pH on top 25 significantly m6A hypermethylated genes in dlPFC of MDD subjects. The data were analyzed using the Pearson Correlation Coefficient. None of the covariates showed any significant correlation.

**Figure S2:** Effect of Race on top 25 significantly m6A hypermethylated genes in dlPFC of MDD subjects (41 Black and 7 White). Each bar plot presents data in the mean ± SEM. The level of significance was determined using an independent-sample ‘t’ test.

**Figure S3:** Effect of positive antidepressant toxicology on top 25 significantly m6A hypermethylated genes in dlPFC of MDD subjects (19 subjects with antidepressant treatment and 30 without antidepressant treatment). Each bar plot presents data in the mean ± SEM. The level of significance was determined using an independent-sample ‘t’ test.

**Figure S4:** Effect of substance use on top 25 significantly m6A hypermethylated genes in dlPFC of MDD subjects (4 subjects with drug abuse history and 45 without drug abuse history). Each bar plot presents data in the mean ± SEM. The level of significance was determined using an independent-sample ‘t’ test.

**Figure S5:** Effect of alcohol abuse on top 25 significantly m6A hypermethylated genes in dlPFC of MDD subjects (18 subjects with positive alcohol toxicity and 24 with negative alcohol toxicity). Each bar plot presents data in the mean ± SEM. The level of significance was determined using an independent-sample ‘t’ test. In 7 MDD cases, alcohol toxicity was not available.

**Figure S6:** Effect of sex on top 25 significantly m6A hypermethylated genes in dlPFC of MDD subjects 30 males and 19 females). Each bar plot presents data in the mean ± SEM. The level of significance was determined using an independent-sample ‘t’ test.

**Supplementary Table Legend**

**Table S1:** This table presents a comprehensive summary of the demographic characteristics of the study participants. The data presented in the table included key information about the factors associated with age, gender, race, and neuropathological assessment details of human study subjects. Each row represents a distinct category of demographic information, and the values are displayed in terms of frequencies, percentages, or means, depending on the variable.

**Table S2:** The table outlines the specific primer sequences utilized in the quantitative polymerase chain reaction (qPCR) assay for the detection and quantification of genes associated with m6A methylation modification. Each row in this table lists the forward and reverse primer sequences.

**Table S3:** The table presents an overview of the m6A methylation profile across gene transcripts in the postmortem brains of individuals with MDD. The data includes information on the specific genes analyzed and the extent of m6A methylation observed. It also provides a comparison between MDD, and control samples based on methylation fold change, highlighting significant and non-significant differences in methylation levels, and many other details, including the genomic coordinates of the transcripts, and prediction of methylation sites on the transcripts.

**Table S4:** The table provides a detailed list of gene transcripts that exhibit hypermethylation in the brains of individuals with MDD. The data includes the specific genes that show elevated methylation levels, expressed in fold change, along with any statistical difference between MDD and control, mentioned as P-value.

**Table S5:** The table provides a detailed list of gene transcripts that exhibit hypomethylation in the brains of individuals with MDD. The data includes the specific genes that show reduced methylation levels, expressed in fold change, along with any statistical difference between MDD and control, mentioned as P-value.

**Table S6:** The table lists gene transcripts with significant hypermethylation in MDD, highlighting their m6A methylation levels expressed as fold change (FC) and the corresponding significance level expressed as m6A P value. These values indicate the degree of methylation variation and its statistical relevance to CNS function in MDD.

**Table S7:** The table presents the relationship between specific m6A methylation levels and corresponding changes in gene expression for 12 select coding gene transcripts. It highlights the m6A methylation status, expressed as fold change (FC), and the associated gene expression changes, indicating how m6A modification influences the regulation of these transcripts. The table also includes statistical significance (P value) for both m6A methylation and expression changes, providing insight into the impact of m6A methylation on gene activity.

**Table S8:** The table presents the Gene Ontology (GO) terms associated with the top significantly hypermethylated gene transcripts in MDD, along with their corresponding P-value and adjusted P-value. The genes listed are determined from each GO term, reflecting their relevance to biological processes and functions.

**Table S9:** This table presents Gene Ontology (GO) terms enriched for synaptic functions, derived from the top significantly hypermethylated gene transcripts in MDD. It includes the Gene Symbol, GO Term ID, GO Term Name, and GO Domain (Biological Process, Cellular Component, Molecular Function) for each significantly hypermethylated gene.

**Table S10:** The table includes Gene Ontology (GO) terms related to inflammatory functions, along with their corresponding P-values and gene names, specifically for the significantly hypermethylated gene transcripts in the MDD brain.

**Table S11:** The table provides a detailed list of gene transcripts that exhibit hypermethylation in the brains of individuals with MDD-S. The data includes the specific genes that show elevated methylation levels, expressed in fold change, along with any statistical difference between MDD-S and MDD-NS, mentioned as P-value.

**Table S12:** The table provides a detailed list of gene transcripts that exhibit hypomethylation in the brains of individuals with MDD-S. The data includes the specific genes that show reduced methylation levels, expressed in fold change, along with any statistical difference between MDD-S and MDD-NS, mentioned as P-value.

**Table S13:** The table presents the list of unique and shared genes identified in MDD and MDD-S brains, highlighting the overlap and distinct gene expression profiles between the two groups. It includes the counts of hypermethylated gene transcripts for individual analysis, along with the corresponding gene symbols. The table categorizes genes based on their presence in either or both analyses, offering insight into the differential methylation profiles and gene expression patterns in MDD and MDD-S brains. In Tab 1, the table describes the unique and overlapping (shared) genes when differentially expressed hypermethylated gene transcripts based on Control vs. MDD were compared with differentially expressed hypermethylated gene transcripts based on Control vs. MDD-NS. It highlights both the distinct and common genes between these two comparisons, along with the counts and gene symbols. In Tab 2, a similar approach is taken, where differentially expressed hypermethylated gene transcripts based on Control vs. MDD are compared with differentially expressed hypermethylated gene transcripts based on Control vs. MDD-S. The table enlists the unique and shared genes in this comparison, providing counts and gene symbols, offering insight into the differential methylation profiles and gene expression patterns across MDD, MDD-NS, and MDD-S brains.

**Table S14:** The table presents the list of unique genes associated with suicide, identified as hypermethylated in the study. It includes gene symbols and provides a glimpse into the potential role of these hypermethylated genes in suicide-related biological processes.

**Table S15:** The table comprises significantly hyper- (a) and hypo- (b) methylated gene transcripts identified in violent suicide completers relative to non-violent cases. It includes gene symbols along with their corresponding log₂ fold change (log₂FC) and p-values, highlighting epitranscriptomic alterations potentially involved in suicide-related biological processes.

**Table S16:** The table presents uniquely hypermethylated 20 gene transcripts identified in violent suicide completers compared to the MDD-suicide cohort. Gene symbols are supplemented with log₂ fold change (log₂FC) values derived from the violent suicide analysis, highlighting unique m6A epitranscriptomic signatures in violent suicide subtypes.

**References**

1. Harrison PJ, Heath PR, Eastwood SL, Burnet PW, McDonald B, Pearson RC. The relative importance of premortem acidosis and postmortem interval for human brain gene expression studies: selective mRNA vulnerability and comparison with their encoded proteins. *Neurosci Lett* 1995; **200**(3)**:** 151-154.

2. Dwivedi Y, Mondal AC, Rizavi HS, Faludi G, Palkovits M, Sarosi A *et al.* Differential and Brain Region–Specific Regulation of Rap-1 and Epac in Depressed Suicide Victims. *Archives of general psychiatry* 2006; **63**(6)**:** 639-648.

3. Salzman S, Endicott J, Clayton P, Winokur G. Diagnostic evaluation after death (DEAD). *National Institute of Mental Health, Rockville* 1983.

4. Spitzer RW G, M, First, MD. *Structural Clinical Interview for DSM-IV (SCID)*. Biometrics Research, New York State Psychiatric Institute: New York, NY, 1995.

5. Benjamini Y, Hochberg Y. Controlling the False Discovery Rate: A Practical and Powerful Approach to Multiple Testing. *Journal of the Royal Statistical Society: Series B (Methodological)* 1995; **57**(1)**:** 289-300.

6. Roy B, Dunbar M, Shelton RC, Dwivedi Y. Identification of MicroRNA-124-3p as a Putative Epigenetic Signature of Major Depressive Disorder. *Neuropsychopharmacology: official publication of the American College of Neuropsychopharmacology* 2017; **42**(4)**:** 864-875.

7. Huang DW, Sherman BT, Tan Q, Collins JR, Alvord WG, Roayaei J *et al.* The DAVID Gene Functional Classification Tool: a novel biological module-centric algorithm to functionally analyze large gene lists. *Genome biology* 2007; **8**(9)**:** R183.
